# Optical Sensors for Bacterial Detection

**DOI:** 10.3390/s23239391

**Published:** 2023-11-24

**Authors:** Olga I. Guliy, Olga A. Karavaeva, Andrey V. Smirnov, Sergei A. Eremin, Viktor D. Bunin

**Affiliations:** 1 Institute of Biochemistry and Physiology of Plants and Microorganisms—Subdivision of the Federal State Budgetary Research Institution Saratov Federal Scientific Centre of the Russian Academy of Sciences (IBPPM RAS), Saratov 410049, Russia; helga1121@yandex.ru; 2 Kotelnikov Institute of Radio Engineering and Electronics, Russian Academy of Sciences, Moscow 125009, Russia; andre-smirnov-v@yandex.ru; 3 Department of Chemistry, M. V. Lomonosov Moscow State University, Moscow 119991, Russia; eremin_sergei@hotmail.com; 4 EloSystems GbR, 13407 Berlin, Germany; vikbun@inbox.ru

**Keywords:** biosensors, optical sensors, colorimetric and fluorescence techniques, surface plasmon resonance, measurement of orientational effects, bacterial cells, detection

## Abstract

Analytical devices for bacterial detection are an integral part of modern laboratory medicine, as they permit the early diagnosis of diseases and their timely treatment. Therefore, special attention is directed to the development of and improvements in monitoring and diagnostic methods, including biosensor-based ones. A promising direction in the development of bacterial detection methods is optical sensor systems based on colorimetric and fluorescence techniques, the surface plasmon resonance, and the measurement of orientational effects. This review shows the detecting capabilities of these systems and the promise of electro-optical analysis for bacterial detection. It also discusses the advantages and disadvantages of optical sensor systems and the prospects for their further improvement.

## 1. Introduction

Bacterial contamination of food and water and the growth of infectious diseases are forcing humanity to look for new pathogen identification methods. In addition, constantly growing threats to personal and territorial security make bacterial detection a priority. Infections are a leading cause of premature mortality, because infectious diseases account for up to 70% of cases [1,2]. The identification and quantification of microorganisms has become key to biodefense, food safety research, diagnostics, and drug discovery. The detection of pathogens and indicator microorganisms in water and food samples is vital to public health and environmental protection. For example, the Department of Disease, Control and Prevention has estimated that each year in the United States, foodborne illnesses cause about 325,000 hospitalizations and 5000 deaths [3].

The accurate detection and identification of bacteria in food and water are very important not only for public safety and health but also for timely and successful treatment. The rapid diagnosis of bacterial infections may ultimately reduce the incidence of diseases and prevent the spread of dangerous epidemics. Traditional laboratory diagnosis methods are lengthy and labor-intensive, and they require expensive equipment and professional personnel. In recent years, substantial progress has been made in the design of accurate, rapid, specific, and cheap methods for the detection of bacteria [4].

An alternative to traditional bacterial detection methods is biosensor systems, which combine the diagnostic capabilities of biomedicine with advances in microelectronics, optoelectronics, and nanotechnology. Of special note are optical biosensors, in which analytical signals are determined by the measurement of physical variables (the intensity of light absorption and reflection, intensity of the object’s luminescence, etc.), rather than by the chemical interaction of the component being determined with the sensitive element. The operating principle of optical sensor systems is based on recording changes in the optical properties of a medium that result from the presence of a detectable biological agent (bacteria or viruses). The optical properties include absorbance (densitometric biosensors), color (colorimetric biosensors), turbidity (turbidimetric biosensors), refractive indices of the medium (refractometric biosensors), and so on.

Although several excellent reviews have been published on the use of optical sensors for bacterial detection [5,6], optical sensor systems based on photonic crystals and ionophores, planar biosensors, fiber optic sensors, and optical sensor systems based on the measurement of orientational effects have not been sufficiently covered in a single review. The purpose of this article is presents an overview of the main directions in the development of optical sensor systems and the use of such systems for bacterial detection.

## 2. Classical Bacterial Detection Methods and Approaches

Usually, bacteria are detected using traditional methods such as culture and colony counting, which in most cases require several processing steps. Traditional bacterial detection methods take at least 72 h to produce a reliable result [7].

Many rapid methods have been proposed to overcome these difficulties, including polymerase chain reaction (PCR) and immunoassays [8,9]. Immunoassay methods, based on the specific antigen–antibody reaction, are used widely for the rapid detection of bacteria. These include

- Enzyme-linked immunosorbent assay.
- Immunomagnetic separation, an analytic tool used to detect bacteria and based on the capture of target bacteria with antibody-coated magnetic beads. It is combined with various detection methods such as PCR [10].
- Methods based on fluorophore-labeled secondary antibodies [11].
- Enzyme-labeled antibodies [12].
- Quantum-dot-labeled antibodies [13,14].

Although immunoassays are quite sensitive, they involve many incubation steps and are, therefore, inconvenient. Biosensors, which combine biological materials such as antibodies, peptides, and nucleic acids with a physicochemical transducer, have become important in the rapid, sensitive, and selective detection of microorganisms.

## 3. Biosensors: Operating Principle and Classification

Three generations of biosensors are available on the market. In the first type, the product comes into contact with the sensor and gives an electric response. In the second type, the sensor includes certain intermediaries between the sensor and the response to obtain a better response. In the third type, the sensor itself gives a response, without an intermediary participating.

Biosensors include a combination of biological detection elements such as a sensor system and a transducer. Different types of biosensors are available depending on the sensor design and the biological material used. On the basis of the transducer system used, biosensors can be classified into optical, electrochemical, thermometric, piezoelectric, and magnetic [15,16,17,18].

In a biosensor, a specific enzyme or a desired biological material is deactivated using a conventional method, and the deactivated material is placed in direct contact with the transducer. In this case, the converter can change the signal associated with it, converting it into electric signals that can be changed and calculated.

**Figure 1:**
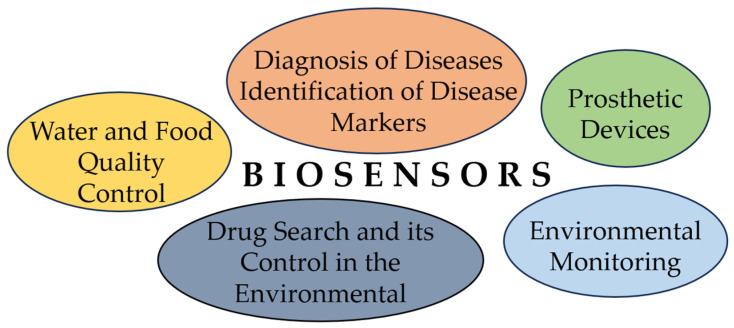
Main applications of biosensors.

Biosensors are applicable in medicine, the food industry, and environmental monitoring, because their sensitivity and stability are better than those of conventional methods. Biosensors are used mostly in environmental pollution monitoring, agriculture, and food processing, and they have become very popular in recent years (Figure 1).

**Figure 2:**
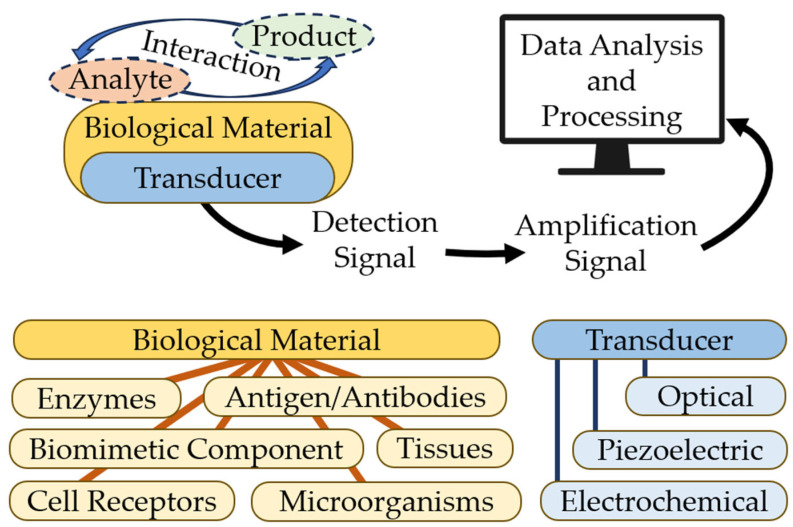
Basic scheme for a biosensor.

Biosensors are characterized by stability, cheapness, sensitivity, and reproducibility. In a biosensor, the core element may be an enzyme, a nucleic acid, or an antibody. The biological element interacts with the analyte, and the biological response can be converted into an electrical signal with a transducer. Biosensors consist of three parts:

- Bioselective element (biological material, (e.g., tissues, microorganisms, organelles, cellular receptors, enzymes, antibodies, and nucleic acids), material of biological origin, or a biomimic material). The sensing element can be made using bioengineering.
- An optical, piezoelectric, or electrochemical transducer, which converts the signal resulting from the interaction of the analyte with the bioselective element into a signal that is easier to measure.
- Associated electronics, primarily responsible for displaying results in a user-friendly manner [18]. Figure 2 shows the basic scheme for a biosensor. Modern technologies in infectious diagnostics and epidemiology have made label-free biosensors increasingly widespread. This allows intermolecular interactions and cellular reactions to be screened, yielding details of the selectivity of bacterial exotoxins, the specificity of antibacterial agents, the antigen–antibody interaction, the kinetics of inflammation, and immunological and serological responses [19,20]. This type of biosensor requires only one recognition element, which simplifies the analysis and reduces its duration and reagent costs. The modern generation of label-free biosensors enables the real-time measurement of the products of biomolecular reactions, continuous recording of data, and kinetic monitoring of the recognition of the ligand–receptor interaction [3,21].

An important advantage of label-free biosensors is that target analytes are detected in their natural form, without labeling or chemical modification, and therefore can be stored for further analysis.

Optical, (piezo)electrical, or (micro)mechanical transducers are promising for recognizing ligand–receptor interaction signals in label-free biosensors used to diagnose infectious diseases. They are enhanced using surface plasmon resonance (SPR) [3], surface-enhanced Raman scattering (SERS) [22,23,24], and nanoparticles [25,26].

## 4. Optical Sensor Systems for Bacterial Detection

The fundamental phenomena underlying the operation of optical sensors are these:

- Absorption (the ability of a substance to absorb optical radiation).
- Reflection (when a stream of light falls on the interface between two media, part of its radiation is reflected back).
- Luminescence (the glow of a substance that occurs after it absorbs excitation energy and is excess radiation, as compared with the thermal radiation of the body at a given temperature).
- Photoluminescence (the emission of photons by a substance that occurs when the substance is excited by electromagnetic radiation in the ultraviolet, visible, and infrared wavelength ranges). Photoluminescence is characterized by absorption and luminescence spectra, polarization, energy yield (the ratio of the energy emitted by a substance in the form of luminescence to the absorbed energy), quantum yield (the ratio of the number of emitted quanta to the number of absorbed ones), and kinetics.

An optical biosensor uses an optical measurement principle, fiber optics, and optoelectronic converters. The term “optrode” is a compression of two terms, “optical” and “electrode”. Aptamers, antibodies, and bacteriophages have highly specific abilities to recognize bacteria and are used as specific biorecognition elements. These elements become important components of the sensor, as they can ensure high specificity and sensitivity of detection [27]. In optical biosensors, antibodies and enzymes are used mainly as transducers, enabling safe, nonelectric, and noncontact sensing. An additional advantage is that they often do not require reference sensors, because a reference signal can be obtained with a light source similar to the sampling sensor. Optical biosensors are divided into two types—direct and labeled.

Biosensors using an optical transducer as the main signal-sensing tool are among the most powerful tools used for bacterial identification. Optical transducers are based on measuring changes in optical properties such as absorption, reflectance, emission, or interferometric patterns, which can be detected with a photodetector. They are immune to electromagnetic interference, capable of remote sensing, and offer a number of advantages, including high sensitivity, direct real-time measurement, and multiplexing capability (detection of multiple analytes at once). Microbial biosensors that detect interactions between microorganisms and target ligands are no exception [28].

**Figure 3:**
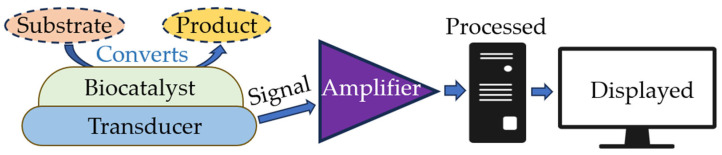
Main components of a biosensor.

In optical biosensors, the analytical signal is determined not by the chemical interaction between the components being measured but by the measured physical parameters, including (but not limited to) the intensity of light absorption and reflection and the intensity of the object’s luminescence. The operating principle of optical biosensors is based on recording changes in the optical properties of the medium that result from the presence of a biological agent. These properties include absorbance (densitometric biosensors), color (colorimetric biosensors), turbidity (turbidimetric biosensors), the refractive index (refractometric biosensors), and other properties. Biosensors are compact analytical devices consisting of two components: a sensitive biological element and a detection system. The detector includes an amplifier, which is known as a signal conditioning circuit, a display unit, and a processor (Figure 3).

Advanced technologies in the design of label-free optical biosensors with an emphasis on the diagnosis of bacterial infections are associated with the development of modern transduction methods (fiber optics and evanescent electromagnetic field systems, surface plasmon resonance, Raman spectroscopy, or interferometry) and new recognition elements (molecular-imprinted polymers) [28].

### 4.1. Colorimetric Sensor System for Bacterial Detection

Colorimetric analysis (from Latin *color* and Greek *μετρώ* ‘measure’) is a physical method of chemical analysis based on determining the concentration of a substance by the intensity of the color of solutions (more precisely, by the absorption of light by solutions). This method has received great development in biomolecule analysis owing to its advantages, including simplicity, stability, and cheapness. ELISA is a classical method for detecting bacteria, which uses an enzyme to catalyze the chromophore and produce a color change in the solution that can be observed with the naked eye or quantified with a spectrometer. This principle is used widely for the rapid identification of bacteria, including in sensor technologies. However, its detection sensitivity is limited by the low extinction coefficient of the organic chromophore. Recently, plasmonic nanomaterials, specifically gold nanoparticles (AuNPs) and silver nanoparticles (AgNPs), have been commonly used in the colorimetric recognition of biomolecules owing to their special optical properties, including high extinction coefficients [29]. For example, a colorimetric method was described that detects Gram-negative bacteria (*Escherichia coli*) on the basis of inhibition of aggregation of AgNPs functionalized with a boronic acid derivative [29]. The degree of color change from yellow to brownish-red depends on the bacterial density. Bacteria were detected in a dynamic range from 5 × 10^4^ CFU mL^−1^ to 1 × 10^7^ CFU mL^−1^, with a limit of detection of 0.9 × 10^4^ CFU mL^−1^. The sensor’s response was fast (20 min). The bacterial density could be determined by the color of the resulting solution in the UV–visible range or with the naked eye.

The capabilities of a colorimetric method for detecting bacteria (*E. coli*) in water that is based on the magnetic separation of proteins of the tail fibers of a T7 phage were shown in [30]. The capture efficiency for *E. coli* ranged from 88.70 to 95.65%, and 10^2^ CFU/mL of *E. coli* could be detected by the naked eye. The authors pointed out that colorimetric changes detected by visual inspection may provide an effective platform for point-of-care detection of *E. coli* in resource-limited areas.

Another study [31] presented a colorimetric sensor system for the detection of *E. coli* O157:H7 in sausage by using magnetic separation and enzymatic signal amplification on a paper disk. The authors pointed out that it is readily applicable to real food (sausage) with an exceptionally low limit of detection (~30 CFU/mL). Problems associated with the nonspecific binding of the sensing components, which lead to high background signal and low sensitivity of colorimetric detection, were overcome by using hyaluronic acid as a blocking agent [31].

Although colorimetry greatly simplifies the detection of bacteria, very often it is characterized by low accuracy and difficulties in determining individual components in the mixture being analyzed.

### 4.2. Fluorescence Sensor Systems for Bacterial Analysis

**Figure 4:**
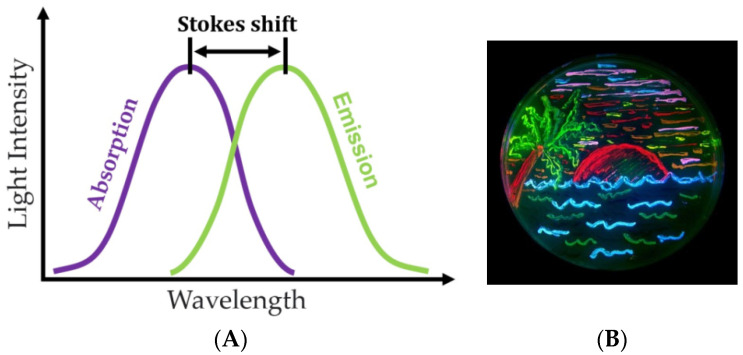
(**A**) Principle of fluorescence analysis and (**B**) Petri dish with bacteria expressing eight fluorescent proteins (figure taken from the Wikipedia website) [33].

Fluorescence has found wide application in applied biological and biomedical research. The essence of fluorescence is the short-term absorption of a quantum of light by a fluorophore (a substance capable of fluorescence), followed by the rapid emission of another quantum, whose properties differ from those of the original one (Figure 4) [32].

Compared with traditional bacterial detection methods, fluorescence probing offers the additional advantage of rapidity of bacterial detection at a relatively low cost.

Among the optical technologies used for bacterial detection, fluorescence sensors have a wide range of applications owing to their hypersensitivity, specificity, and accuracy [34]. Three main strategies for bacterial detection with a fluorescence sensor can be distinguished:

(1) Fluorescence imaging to identify the bacteria;
(2) Enhancement or changing of the method’s characteristics when targets are combined;
(3) Quenching of fluorescent signals through energy receptors, such as quenching agents [35].

The main trends in the development of fluorescence sensors for bacterial detection are these:

- Use of appropriate fluorescent materials;
- Use of DNA, bacteria, and metabolites as the response targets;

Each fluorophore has its own specific absorption and emission spectra, depending on the structure of the molecule and its environment. Initially, researchers used traditional fluorescent dyes as fluorescent agents. However, these dyes have significant disadvantages, including a high background, a narrow excitation spectrum, a wide and asymmetric fluorescence spectrum, and ease of photobleaching, which limit their application in the biomedical field. It is expected that with rapid developments in nanomaterials and nanotechnology, new fluorescent nanomaterials will gradually replace traditional luminescent materials owing to their high fluorescence quantum yield and excellent light stability. The main advantages of using fluorescent nanomaterials for bacterial detection are their small size, high adsorption capacity, and high surface reaction activity. Bacteria can simultaneously combine with multiple nanoparticles, thus permitting a higher fluorescence intensity to be achieved in the labeled target bacteria. Owing to the high stability of fluorescence, bacteria can be monitored in real time from minutes to hours. In addition, multicolored fluorescent nanoparticles can be used to simultaneously detect different strains. The toxicity of fluorescent nanoparticles is strongly reduced after their surface is coated with inert substances.

When used in sensors, fluorescent nanomaterials can be conjugated with biomolecules (enzymes and nucleic acids), which results in a high sensor performance. In recent years, several new materials have been developed, including QDs, CDs, UCNPs, and MOFs. These materials possess improved chemical and physical properties and can be used to identify, transmit, and amplify signals for bacterial detection [35,36,37,38,39]. The potential application of two-level column-like 3D nanofilms in optical sensing technologies was described in [40].

Several different modifications of fluorescence assay methods for detection have been described, as follows:

*Escherichia coli*: detection limit, 1.0 CFU/mL [41]; detection limit, 4.0 × 10^1^ CFU mL^−1^; concentration range, 1.3 × 10^2^ CFU mL^−1^–6.5 × 10^4^ CFU mL^−1^ [42]; concentration range, 1.3 × 10^2^–1.3 × 10^8^ CFU mL^−1^; detection limit, 3 CFU mL^−1^ in [43]; concentration range, 10–10^5^ CFU/mL; detection limit, 8 CFU/mL in [44]; detection limit, 50 cells/mL [45]; concentration range, 10^3^–10^5^ cells mL^−1^ [46]; concentration range, 10^3^–10^6^ CFU mL^−1^ [47]; and detection limit, 10^5^ cell/mL [48].

*Salmonella typhimurium*: detection limit, 50 cells/mL [45]; concentration range, 10^3^–10^5^ cells mL^−1^ [46,49]; concentration range, 10^3^–10^6^ CFU mL^−1^; detection limits, 100 and 138 CFU mL^−1^ in a fresh-cut vegetable washing solution and a lettuce sample, respectively [50].

*Staphylococcus aureus*: detection limit, 10^5^ cell/mL [48]; and concentration range, 1 × 10^3^–1 × 10^11^ CFU mL^−1^; detection limit, 1 × 10^3^ CFU mL^−1^ [51]; concentration range, not given [52]; concentration range, 47–4.7 × 10^7^ CFU mL^−1^; and detection limit, 10.7 CFU mL^−1^ [53].

In *Klebsiella pneumonia,* the detection limit is 10^5^ cell/mL; in [48] *P. aeruginosa*, *B. cereus* 10^3^–10^5^ cells per mL in [46]; etc.

**Figure 5:**
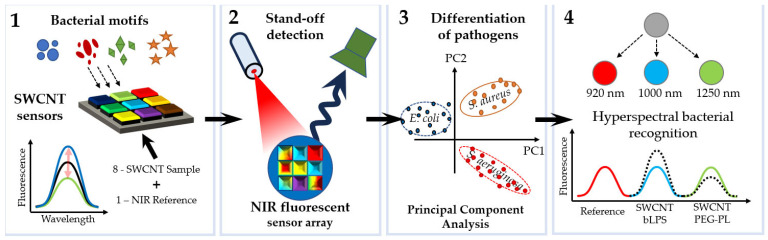
Remote detection of pathogens. (1) Multiple nanosensors based on NIR fluorescent single-walled carbon nanotubes are synthesized in such a way that they change their fluorescence signal in response to bacterial metabolites and virulence factors (cell wall components, iron chelating molecules, and secretory enzymatic activity). (2) Eight fluorescent nanosensors and one NIR fluorescent reference are incorporated into a polyethylene glycol hydrogel array that is remotely monitored in the NIR. (3) Bacteria growing on top of this hydrogel release molecules that change the (spatial) sensor array fingerprint, which allows one to differentiate between important pathogens. (4) By using chirality-purified single-walled carbon nanotubes, multiple sensors can be spectrally encoded and used for hyperspectral differentiation of important bacteria such as *S. aureus* [54], with modifications.

An important direction in the development of fluorescent sensors is the so-called remote detection of pathogens. For example, near-infrared (NIR) fluorescence nanosensors were used for the remote fingerprinting of clinically important bacterial detection [54]. Multiple nanosensors based on NIR fluorescent single-walled carbon nanotubes were synthesized in such a way that they changed their fluorescence signal in response to bacterial metabolites and virulence factors (cell wall components, chelating iron molecules, and enzymatic activity). Using several sensors with different selectivities allowed the authors to clinically distinguish between relevant bacteria on the basis of their metabolic fingerprints. Multiplexing was achieved through spatial or spectral coding, which emphasizes the potential for remote pathogen detection (Figure 5) [54].

Hydrogels were exposed to clinical isolates of six important bacteria, and remote (≥25 cm) NIR imaging allowed the authors to identify and distinguish between the bacteria. Sensors were also spectrally encoded (900, 1000, and 1250 nm) to differentiate between the two major pathogens, *P. aeruginosa* and *S. aureus*, and penetrate into tissue (>5 mm). In the future, the remote near-infrared detection of bacteria will enable faster diagnosis and individualized antibiotic treatment, ultimately leading to better clinical outcomes and lower mortality rates.

A fluorescence and an optoelectric recording device were combined for automatic real-time photoelectric microbial sensor analysis [55]. The system enabled the ultra-sensitive (1 CFU/mL) and specific (99%) detection of *E. coli* and *P. aeruginosa*. This system can possibly be extended to detect other microorganisms.

Recent advances in fluorescent dyes capable of detecting bacteria and fluorescent sensor assay strategies for identifying bacteria, including specific interactions with the bacterial cell wall, have been described in the reviews [38,56,57].

**Figure 6:**
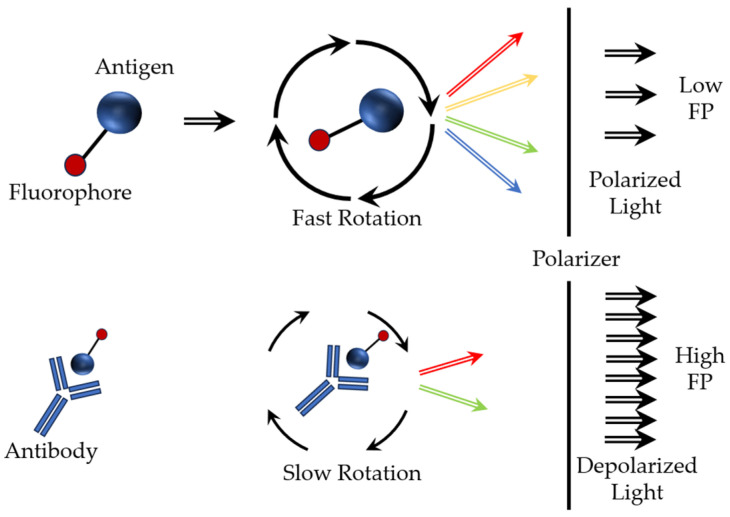
Basic operating diagram of the fluorescence polarization immunoassay.

A separate mention should be made for fluorescence polarization immunoassay, based on the fluorescence and polarization of light [58]. Fluorescence is the process of transition of an electron from an excited singlet state to the ground state. This process is accompanied by a release of energy in the form of radiation with a certain wavelength. If fluorescent molecules are excited by plane-polarized light, the emitted radiation must be polarized in the same plane, but only if the molecule is stationary. If the molecule rotates while in an excited state (the period between the absorption of light and the emission of fluorescence), then fluorescence is emitted in a plane different from that used for excitation. This leads to depolarization of the emitted light (Figure 6). For small molecules that undergo rapid Brownian rotation in solution (e.g., a fluorescently labeled low-molecular-weight antigen (tracer) labeled with a fluorescent label), the fluorescence polarization value is low. Larger molecules (e.g., fluorophores) bound to antibodies are less mobile in solution and have higher fluorescence polarization values (Figure 6). The molecular volume of a fluorescently labeled substance can be changed as a result of cleavage (dissociation), binding to another molecule, or conformational changes.

The history and application prospects of this method, including its use in the diagnosis of diseases, were described in [59]. The capabilities of fluorescence polarization analysis using fluorescein-labeled ESAT-6 protein were shown in the detection of antibodies to *Mycobacterium bovis* in bovine serum [60] and antibodies against *Brucella melitensis* in goat milk [61].

Fluorescence polarization immunoassay is highly specific, sensitive, and productive, and it is simpler, faster, and more reproducible than ELISA. Equilibrium for the antigen–antibody reaction in solution is established within 1–2 min, and the fluorescently labeled tracers remain stable for many years of storage. In addition, the method can be easily automated; in particular, Ellie (Milwaukee, WI, USA) [62] produces the portable polarization fluorometer Sentry-300, which is used widely in diagnostic laboratories and in non-laboratory settings. Despite these advantages, fluorescence polarization immunoassay has several limitations: specifically, it is applicable only to the determination of low-molecular-weight compounds. Because the analysis does not involve separation of components, the proteins, endogenous fluorophores, inhibitors and activators of protein binding, and other substances present in the reaction mixture will always affect the results of the determination.

With account taken of the great progress made in nanomaterials and sensor research, fluorescence-based sensors for bacterial detection show great promise and will play a key part in bacterial detection.

### 4.3. Chemiluminescence

Chemiluminescence (CL) is another optical method alternative to the colorimetric approach, which is triggered by the transition of an atom or a molecule from an excited state to a steady state with the emission of photons as a by-product of a chemical reaction [63]. CL-using biosensors are typically based on enzyme indicators able to catalyze CL in the presence of a suitable substrate. The measurement of the produced photons can be related to the concentration of the target analyte, allowing quantitative analysis to be conducted, but this implies the use of an external detector capable of detecting luminescent signals. Currently, there is a wide range of miniaturized and compact CL detectors that can be used in portable analytical devices [64].

CL biosensors have been developed and widely applied to mammalian cells and the study of intracellular signaling networks. With respect to bacteria, biosensors relied heavily on fluorescence-based systems to quantify signaling molecules, but in challenging environments, such designs can face problems owing to their dependence on ambient light. To circumvent these problems, ratiometric CL biosensors were developed to study cyclic di-GMP, the key bacterial secondary messenger [65]. For combining the advantages of electrochemical and optical bacterial detection, electrochemiluminescence is widely used for routine analytical operations in the laboratory. It is triggered by an electrochemical stimulus on a specific molecule and ensures high control of the emitted light, a low background signal, and high sensitivity. These aspects make electrochemiluminescence particularly suitable for the development of portable biosensor devices [66]. Compared with fluorescence, electrochemiluminescence does not require an excitation light source, thereby avoiding autofluorescence or a diffuse light background. Moreover, the use of potential to initiate and regulate the output signal provides electrochemiluminescent sensors with high repeatability and accuracy [67].

### 4.4. Sensors Based on the Surface Plasmon Resonance

Currently, the best developed optical biosensors are those based on changes in the direction of propagation of a light flux through an optical fiber or a triangular prism coated with a thin metal film. Biosensors of this type are based on surface plasmon resonance (SPR). The intermolecular interaction is detected by the change in the refractive index of the medium as a result of the formation of an antigen–antibody complex on the resonance layer of a measuring cell or a flow cell [68,69]. SPR-based biosensors are sensitive and use a special mode of electromagnetic welding, the surface plasmon polariton, to detect the binding of the analyte molecules from a liquid sample with biomolecular recognition elements on the sensor surface [70].

**Figure 7:**
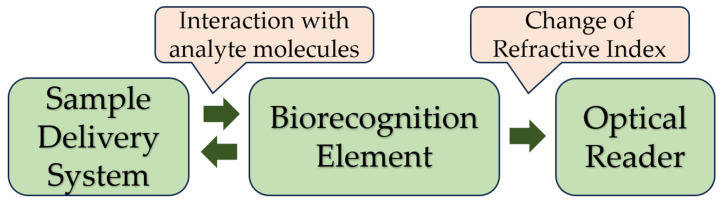
Principal components of an SPR affinity biosensor.

SPR-based optical affinity biosensors are some of the most advanced [71]. Their ability to control the interaction between a molecule immobilized on the sensor surface and an interacting molecular partner in solution has made SPR sensors very powerful for analyzing biomolecular interactions. In recent years, SPR biosensors have been increasingly used to detect chemical and biological substances related to medical diagnostics, environmental monitoring, and safety. SPR biosensors consist of three main subsystems: sensor equipment (optical reader), a biorecognition element, and a sample preparation and delivery system (Figure 7).

**Figure 8:**
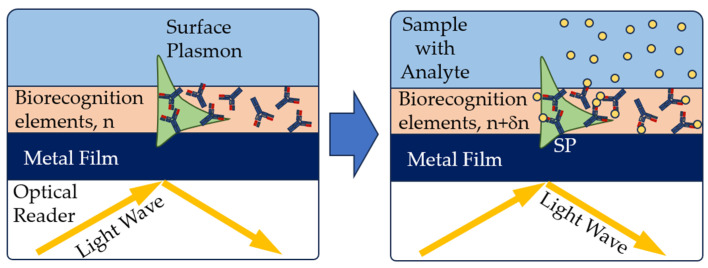
Principle of operation of SPR affinity biosensors [71], with modification.

In the optical reader of the SPR sensor, a light wave excites a special mode of the electromagnetic field called a surface plasmon. The surface plasmon propagates along a thin metal film, and its field probes the medium adjacent to the metal surface. Any change in the refractive index near the metal surface leads to a change in the velocity of the surface plasmon. This change can be determined by the characteristics of the light wave associated with the surface plasmon. Biorecognition elements are immobilized on the metal surface. If a liquid sample comes into contact with the sensor surface, the analyte molecules are captured by the biorecognition molecules (Figure 8). This binding leads to a refraction index change near the sensor surface, which is recorded by the sensor [72].

A surface plasmon is an electromagnetic wave propagating along the boundary between a dielectric and a metal [73]. Most SPR sensors are based on the Kretschmann attenuated total reflection configuration.

**Figure 9:**
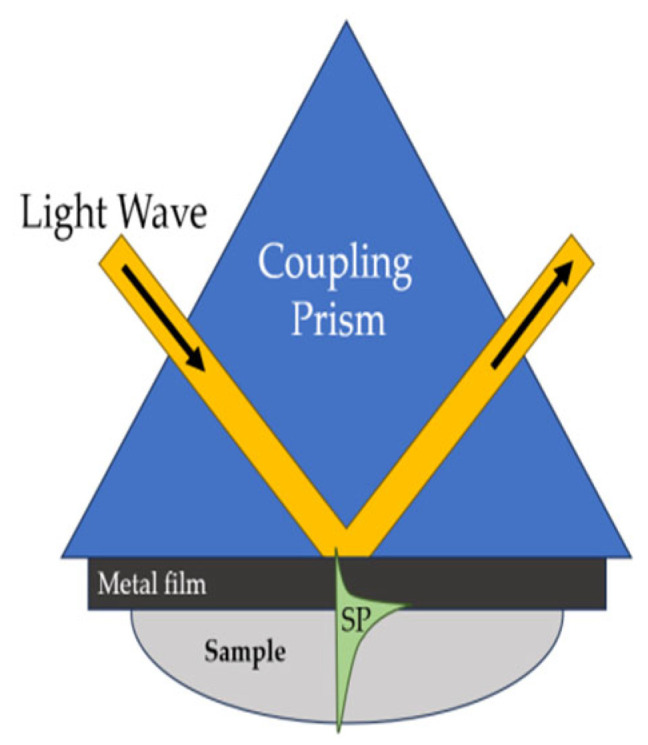
Surface plasmon excitation in the Kretschmann geometry of the attenuated total reflection method [71], with modification.

In this geometry, the light wave passes through a prism with a high refractive index and is completely reflected from the base of the prism, covered with a thin gold film (Figure 9). Light tunnels fleetingly through a thin metal film and can excite a surface plasmon at the outer edge of the metal. The interaction of the incident light wave with the surface plasmon is accompanied by a transfer of energy and leads to a decrease in the intensity of the reflected light wave. Because anchoring occurs only over a narrow range of incidence angles (or wavelengths), surface-plasmon excitation causes a narrow dip in the angular (or wavelength) spectrum of the reflected light. On the basis of these characteristics, the reflected light wave is measured with the SPR sensor equipment [71].

In the field of optical or photonic sensor technology, other approaches are based on plasmonic resonator structures [74], optoplasmonics [75,76], met surfaces, strip-waveguide ring resonators, and strip-waveguide resonators [77,78,79], which offer attractive possibilities for bacterial detection.

Photonic crystal plates such as those used in photonic crystal-enhanced microscopy are a special form of structured surfaces used in sensors [80,81,82]. Measurement methods based on some of these structures include surface-enhanced Raman scattering (SERS) [83,84] and surface-enhanced infrared absorption (SEIRA) spectroscopy [85,86,87]. SEIRA allows identification of specific biomolecules or classes of biomolecules by generating specific chemical resonances at mid-infrared wavelengths in organic materials. Photonic crystal-enhanced microscopy typically generates spectrally encoded images, such as color coding of the depth of penetration of light into a cell or local changes in the refractive index of the cell, for which various spectroscopic and spectrometric methods are used [85,86,87].

An obvious alternative to the refractive index approach is to use a fluorescent tag to analyze the bioanalyte of interest. In particular, fluorescent labeling has been used in the “classical” immunosensory competition reaction between antibodies and antigens, in which the presence of specific antigens in the analyte suppresses the fluorescence of identical antigens that have been fluorescently labeled and added to the analyte. For example, Mathias et al. [88] showed that, as compared with a plain glass substrate situation (i.e., glass slide), there is a noticeable (60-fold) increase in fluorescence from the fluorophore cyanine-5 deposited on a streptavidin-conjugated photonic crystal substrate.

The rapid development of SERS in the past 20 years has been facilitated by advances in the targeted synthesis of nanostructured materials and the development of and improvements in Raman spectrometers. SERS offers unprecedented possibilities for lowering the detection limits for relevant analytes and their multiplex determination. SPR biosensor technology has been successfully used to detect various analytes such as proteins, drugs, DNA, and microorganisms [89]. This technology shows promise for the detection of specific pathogens through the use of nanosized materials, which ensure the performance and high selectivity of SPR-based sensors [89].

**Figure 10:**
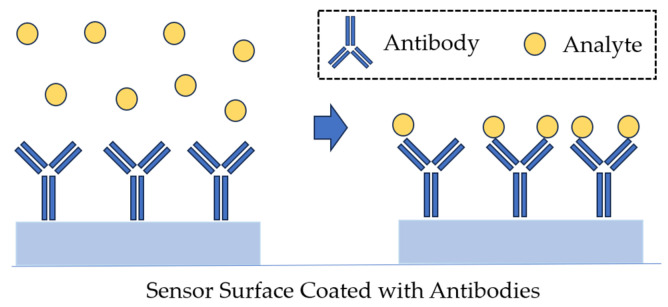
Direct detection, as used in SPR biosensors.

SPR-based immunosensors coupled with a specific antigen–antibody reaction detect bacteria in a sensitive, specific, rapid, and label-free manner. The integration of new technologies in materials science and molecular biology with the SPR should permit the design of biosensors with detection limits compatible with those offered by traditional methods [24]. In a direct detection format, the analyte molecule in the sample binds to biorecognition elements immobilized on the sensor surface (e.g., antibodies; Figure 10), and the sensor’s response is proportional to the concentration of the captured analyte [71].

**Figure 11:**
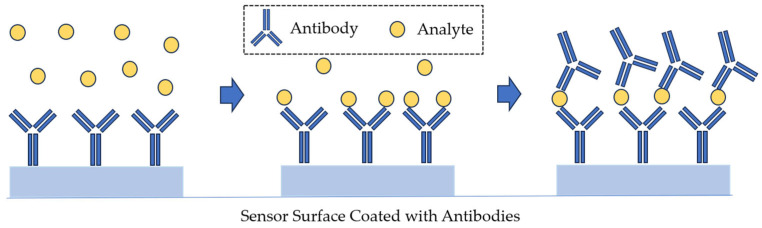
Sandwich assay detection, as used in SPR affinity biosensors.

In the direct detection format, the flow cell of the SPR sensor is initially loaded with a buffer (both sensing and reference channels). As soon as the base line is established, the sample is injected and incubated with the sensor surface for a specified period. The lowest detection limits of the direct detection sensors can be improved using sandwich assay. In such an assay, secondary antibodies bind to the analyte on the sensor surface that has been previously captured by antibodies. For example, the detection limit of an immunosensor for *E. coli* O157:H7 decreased from 10^6^ to 10^3^ CFU/mL owing to the sandwich assay procedure [90]. Sandwich assays are typically used not only to improve the detection limit beyond what is available in the direct detection format but also to improve specificity and detection. This approach is also suitable for detection in complex matrices [91]. In a sandwich assay, a primary biorecognition element immobilized on the sensor surface captures the analyte from the sample, and a secondary biorecognition element binds to the previously captured analyte [71] (Figure 11).

**Figure 12:**
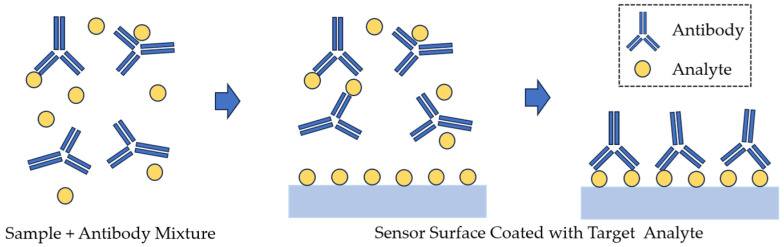
Binding inhibition assay detection, as used in SPR affinity biosensors.

Competitive assays are typically used to detect low concentrations of analytes whose binding to surface-immobilized recognition molecules does not produce a measurable sensor response. In the binding inhibition assay (Figure 12), analyte molecules or their derivatives are fixed to the sensor surface [71].

A known number of biorecognition elements are incubated and allowed to bind to the analyte in the sample. Then, the mixture is washed off the sensor surface, and unreacted biorecognition elements are fixed. In this case, the sensor response is inversely proportional to the analyte concentration. This approach increases the sensitivity of the assay but also increases the cost and time of analysis owing to the use of secondary antibodies and multistep manipulations.

SPR-based immunosensors have great potential for the rapid detection of microorganisms in food and water samples. However, the detection limits achieved with these sensors cannot compete with those offered by traditional methods. Efforts have been made to increase the sensitivity of immunosensors on the basis of the sandwich assay [91,92,93] and the subtractive inhibition assay (SIA) [94,95].

To develop new biomolecular recognition elements with higher stabilities and higher affinities for target bacteria, researchers have recently integrated phage display with molecular imprinting in SPR biosensors [96]. Bacteriophages are a useful alternative to antibodies in the detection of bacteria. For example, *S. aureus* was detected with an SPR sensor by using a lytic phage as the recognition element, and the detection limit was 10^4^ CFU/mL [97].

Phage display technology is another tool for developing single-chain antibodies to bacteria. Phage-like antibodies have advantages such as in vitro production, a low cost, a low molecular weight, and high stability [98]. Phage display antibodies have been used to detect *Listeria monocytogenes* in an SPR biosensor (detection limit, 2 × 10^6^ CFU/mL), which indicates the promise of non-antibody recognition elements [98].

In the past few decades, SPR-based sensors have been used for the simultaneous detection of multiple analytes, but they are limited by the number of analytes. The use of multianalyte sensors will depend on the needs of specific applications. Primary applications of such sensors include pharmaceutical research (high-throughput drug screening systems), medical diagnostics (high-throughput diagnostic tools), food safety (systems for the rapid detection of pathogens and foodborne agents), and security (devices for the early detection and identification of biological warfare and chemical agents) [70].

In SPR-based sensors, molecular imprinting technology is as effective as antibody- or phage-based technology. Idil et al. (2021) described a variation in the SPR-based sensor that uses micro-contact imprinted chips to detect *S. aureus* within the range 1.0 × 10^2^–2.0 × 10^5^ CFU/mL. The authors showed that this platform is applicable to actual samples (milk) [99]. A few commercial SPR biosensor systems (Biacore, Uppsala, Sweden) for bacterial detection are described in [100].

**Figure 13:**
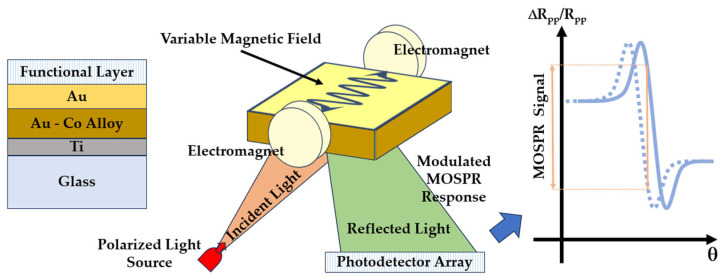
Principle of operation of MO–SPR sensors.

As already mentioned, SPR-based detection methods are not always highly sensitive. Sensitivity can be increased by introducing magneto-optical SPR (MO–SPR). This method uses the magneto-optical Kerr effect (TMOKE), which results from the use of a magnetic field perpendicular to the plane of propagation of incident *p*-polarized light [101,102]. When the TMOKE is combined with plasmonic effects, new SPR-based detection configurations emerge, leading to the MO–SPR method (Figure 13). With such a change in the refractive index, the sensitivity of the method can be improved by 10 or even 20 times.

Chen et al. (2021) used a magneto-optical biochip based on the Cotton–Mouton effect of γ-Fe_2_O_3_@Au core/shell magnetic nanoparticles to determine spike glycoprotein S in the diagnosis of SARS-CoV-2. The sensor can be tailored to be used for diagnosing diseases other than SARS-CoV-2. Importantly, the analysis takes 50 min and the detection limit of our method for the spike glycoprotein S of SARS-CoV-2 is estimated to be as low as 0.27 ng/mL (3.4 pM) [103]. The prospects for such sensor systems have been described in [101,102].

### 4.5. Fiber Optic Sensors

**Figure 14:**
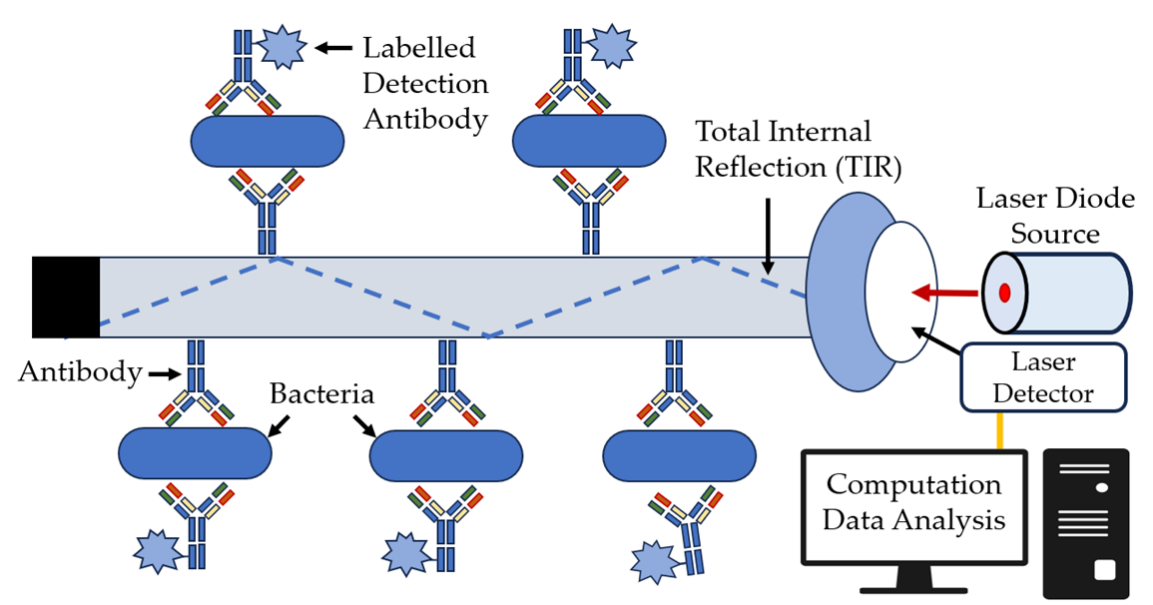
Bacterial detection with a fiber optic biosensor [104], with modifications.

The use of optical fibers as bio- and immunosensors is promising, because they are cheap and offer rapid and easy analysis. Typically, radiation of various wavelengths of the infrared (IR) spectrum is supplied through the fiber. Fiber optic biosensors use the property of the total internal reflection of light as it passes through a waveguide and creates an evanescent wave boundary at the waveguide surface. Antibody- or immunoassay-based fiber optic sensors provide increased sensitivity, selectivity, and speed, as compared with conventional immunoassay methods [104]. The general diagram of such a sensor is shown in Figure 14.

Optical biosensor fibers can be combined with various spectroscopic methods. Chemi-, bio-, and electroluminescent sensors provide the very sensitive detection of specific substrates. Because many microorganisms have specific IR spectra, fiber IR spectroscopy can be used to detect changes in the properties of biological objects. Immobilization of antibodies inside the fiber ensures specific indication.

Antibody-based fluorescent waveguide biosensors are typically used in the detection of bacteria. First, a specific fluorescently tagged antibody (e.g., Cy-5 or Alexa-Fluor 647) covalently binds to an optical fiber that captures the bacteria of interest, and the detecting antibody specifically binds to the bacteria (Figure 14). Portable sensors such as the Analyte 2000 and RAPTOR from Research International (Monroe, WA, USA) are used widely for such applications [105,106,107,108]. These systems allow the qualitative detection of the target object, and the signal is proportional to the antigen or hapten amount present in the sample [109].

A separate direction in the development of optical fiber sensors is Mach–Zehnder Interferometers (MZIs), which create a picture of interference fringes inside the attenuation bands when bacteria are detected. For example, a sensor system was described that is based on label-free optical fiber made of two gratings of the same type (intergrating space, 1 cm) and immobilized antibodies against *E. coli*. The detection limit was 7 CFU/mL [110]. Another study presented the potential of sensors based on long-period, label-free fiber arrays for detecting bacteria [111].

Polyelectrolyte functional coating is used for the specific detection of *S. aureus*. The detection limit for an *E. coli* sensor was 100 CFU/mL, with MS2 phage as the receptor, and that for a sensor based on peptide aptamers was 10 CFU/mL [111]. A dual-taper microfiber-based MZI biosensor for the detection of *S. aureus* and the use of porcine immunoglobulin to functionalize the microfiber for the detection of *S. aureus* bacteria were reported. The maximum wavelength shift of 1.408 nm was achieved when the biosensor was immersed into an *S. aureus* suspension (cell density, 7 × 10^1^ CFU/mL). The detection limit was as low as 11 CFU/mL [112].

Immunosensors have been developed on the basis of optical fiber to detect the capsular antigen of the plague microbe [113,114,115] and antibodies to it [116].

Fiber optic biosensors are reflected in the RAPTOR™ detection system (Rapid Automatic and Portable Fluorometer Assay System; Research International, USA) [105,117,118]. The RAPTOR™ biosensor allows one to simultaneously detect more than four types of dangerous agents, including *Francisella tularensis*, ricin, and *Staphylococcus* enterotoxin [105]. A total of 203 blind samples were tested for staphylococcal enterotoxin B, ricin, *Francisella tularensis*, and *Bacillus globigii*. The sensitivities obtained were 10 ng/mL, 50 ng/mL, 5 × 10^5^ CFU/mL, and 5 × 10^4^ CFU/mL, respectively. The advantages of optical biosensors include high sensitivity, a short response time (1–3 min), a non-contact nature, and the low influence of electric noise.

Fiber optic biosensors can detect microorganisms without the use of specific molecules. They are easy to use, small, and cheap, and so they are most promising in the signal detection of biological pathogens.

In recent years, the use of fiber optic sensors for the detection of pathogenic bacteria and their toxins has increased. For example, such sensors have been successfully used to detect as low as 10^3^ CFU/mL of pure-cultured *E. coli* O157:H7 cells [107]. The *Listeria monocytogenes* sensitivity threshold was about 4.3 × 10^3^ CFU/mL in [106] and 5 × 10^5^ CFU/mL in [108]. The *Salmonella* detection limit was ≥10^5^ CFU/100 mL [118], and that for *Streptococcus* was not given by the authors [119].

Geng et al. (2006) [109] detected *E. coli* O157:H7 with an initial inoculation of 1 CFU/g ground beef after only 4 h of enrichment. By using RAPTOR™, 5 × 10^5^ CFU/mL of *Salmonella typhimurium* was detected in sprout rinse water [120]. Geng et al. (2004) reported a fiber optic method for *L. monocytogenes* detection with a limit of 10^3^–10^4^ CFU/mL in hot dogs and bologna, and this was later confirmed with RAPTOR™ [106].

Combining a fiber optic biosensor with PCR increases the detection sensitivity and strongly improves the detection rate—from the 10 h required for conventional fiber optic sensors to 2 h [104].

Separate mention should be made of an optical fiber sensor based on quantum-dot immunofluorescence for *S. aureus* detection (detection limit, 10^3^–10^4^ CFU/mL). The sensor combines the specificity of the antigen–antibody interaction with the stability of quantum-dot fluorescence [121]. The application of label-free fiber optic biosensors in bacterial detection has been described in [5,122,123].

It is also necessary to mention optical waveguide light spectroscopy [124] and interferometric methods, for example, those based on the Mach–Zehnder interferometer [125,126], which can detect bacteria or other pathogens in liquid samples. A common feature of these methods is that they use specific adsorption on the waveguide surface and measure the corresponding changes in the intensity of the directional light with photodetectors and carried out quantitative measurements by observing the light scattered by living *E. coli* located in the vicinity of the waveguide with a detection limit of 10^2^ CFU × mL^−1^ [127].

Recent advances in nanomaterial-based fiber optic biosensors, with the possibility of their practical application, are presented in the review [127,128,129,130]. Binding nanomaterials to biological receptors improves their sensitivity, detection rate, specificity, and sensor response time, thereby providing many of the performance benefits of fiber optic biosensors. These advantages make fiber optic sensors ideal for commercialization.

### 4.6. Optical Sensors Based on Ionophores and Planar Biosensors

A special place among the active substances that determine the sensor selectivity is occupied by ionophores. These are neutral or charged substances that selectively bind to the analyte. A traditional example of an ionophore-based optode is a hydrophobic volumetric optical sensor. This is a plasticized poly(vinyl chloride) matrix or another polymer matrix with similar properties that contains lipophilic active components (ionophore, an indicator that changes its optical properties). The analytical signal in optodes is a change in a certain optical property (light absorption, luminescence intensity, etc.) in response to the quantitative content of the analyte in the contacting sample. The optical signal can be recorded even with a color camera, and information about the analyte content in the solution can be obtained using digital image processing. It is also possible to record changes in optical properties with spectral instruments (a spectrophotometer for recording the absorption of visible light, a spectrofluorimeter for recording luminescence, etc.). Although optodes are promising in the making of non-invasive flexible sensor platforms for use in situ, their capabilities have not been sufficiently developed. Optical sensor membranes can be placed on optical fibers or on planar substrates and can also be manufactured in the form of micro- and nanoparticles. Optical sensor platforms based on planar substrates make it possible to integrate sensors selective for different analytes (multianalyte sensor arrays) on a single substrate. Optodes, especially luminescent optodes, are characterized by high-signal amplitudes. This allows them to be used for visualization and sensing to solve practical problems in biology and biotechnology. For example, by immobilizing optodes inside an agarose membrane (a medium for bacterial growth), Wang et al. [131] visualized the acidity of the medium during the life cycle of microorganisms.

Current optode research is active and is a promising and rapidly developing direction of non-destructive analysis [132,133,134]. Sensor arrays of optodes have been described that can be used for in situ analysis. These include well-proven planar arrays of optodes, which make it possible to determine changes in the biofilm oxygen concentration online using the detection of luminescence decay [135,136]. A planar optical waveguide was proposed for gas and humidity sensing by measuring the change in the refractive index of the material in the waveguide cap [137]. This has led to tremendous progress in optical technology. Guided wave sensors are used to measure humidity, heavy metals, and biological objects, including microbial cells [138].

**Figure 15:**
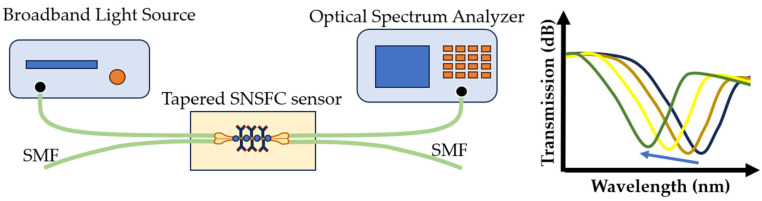
Experimental setup of the sensor and transmission versus wavelength for *S. aureus* detection.

The combination of evanescent field sensing-based measurements and optical phase difference has led to the development of an integrated planar optical waveguide interferometry biosensor. The signal created by interfering fields is detected at the sensor output and is related to the analyte concentration [139]. This biosensor requires a few samples to measure binding interactions, is easy to use, and is cheaper than other optical biosensors. Other studies used a similar sensor with a structured fiber optic coupler to detect *S. aureus* with a detection limit of 3.1 CFU/mL [140] (Figure 15). Such a biosensor is applicable in the analysis of cellular processes [141]. This method is also very useful for detecting avian influenza virus [142].

Considerable efforts have been made to make biosensors based on the evanescent field interaction. Horváth et al. [123] described a planar grating-coupled optical waveguide sensor that uses evanescent waves for bacterial detection. The waveguide design ensures an increased penetration depth into the sample, making the sensor suitable for detecting micrometer-sized biological objects. Using the sensor, the authors monitored the adhesion of *E. coli* K12 to the sensor surface.

**Figure 16:**
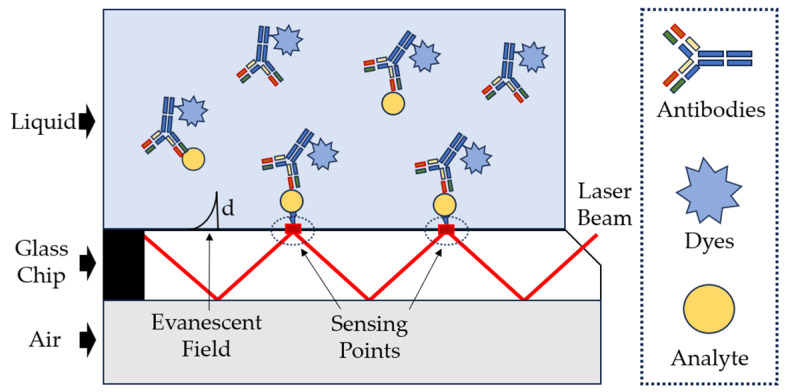
Scheme for an evanescent-wave planar optical waveguide chip [140], with modification.

Of note are biosensors based on evanescent wave fluorescence, in which biological recognition and the manifestation of subsequent binding to the analyte occur within the boundaries determined by fleeting waves. Detection occurs on the basis of excitation of fluorescent molecules near the surface (Figure 16), which helps reduce background signals arising from sample analysis [102,143].

An example of a possible practical application of a waveguide-based optical sensor is the miniature PEGASUS device, which can detect toxins, bacterial signatures, viral signatures, and much more in samples such as blood, water, cerebrospinal fluid, and food. PEGASUS does not require trained personnel or laboratory equipment and can easily be used in remote areas of the world. The device can differentiate between bacterial and viral signatures, ensuring the selection of appropriate treatment to improve patient outcomes and reduce the spread of antimicrobial resistance.

Detection occurs in two main steps. First, the sample is processed in a microfluidic device, which requires only a small sample volume, and second, the processed sample is loaded into a miniature detecting sensor. For bacterial infections, it takes the researcher 15–30 min to distinguish between Gram-positive, Gram-negative, and uncertain sources without prior knowledge of the type of infection. To create a sensitive field, a laser at a critical angle of incidence is connected to a planar waveguide, and the total internal reflection of light occurs between the layers of the waveguide owing to their different refractive indices. This causes an evanescent field to be emitted from the wave guide surface, where the fluorescent molecules are detected. The sensor includes a built-in, low-hands sample processing device designed to ensure that every sample is of the quality needed for detection [144].

### 4.7. Photonic Crystal Biosensors

Of particular interest are biosensor devices using photonic crystals, the operating principles of which can be found in [145]. The spatial periodicity of optical properties is a characteristic feature of photonic crystal structures. The optical waves measured in these sensors are tangible manifestations of Maxwell’s equations, and frequency or wavelength plays a direct role in relation to the characteristics of the medium through which light or, more generally, electromagnetic waves propagate. The transport of information and energy in a photonic crystal is controlled by the total group velocity of Bloch modes at a given frequency of the electromagnetic wave (that is, light at optical frequencies) that impinges on the photonic crystal medium. The speed of the Bloch mode in a periodic medium is determined by coherent multiple (Bragg) scattering on a regular arrangement of photonic “atoms” of a photonic crystal. Threm et al. [146] reviewed the application of various types of photonic crystal structures for use as biosensors. Fan et al. [21] examined optical biosensors that use the principles of cell-free analysis, i.e., changes in the local refractive index associated with the specific binding of the target biomaterial, and not, for example, fluorescent labeling.

Optoelectronics has seen many potential applications of photonic crystals in biosensors [147]. A fundamentally new class of optical biosensor is a sensor based on photonic crystal fibers with a hollow core [28]. The operating principle of photonic crystal waveguides is based on the detection and identification of biological objects by using the spectra of light passing through a hollow core filled with the material under study in the wavelength range 200–1100 nm. Photonic crystal waveguides have channels that can be filled with working solutions. The total working surface area of a photonic crystal fiber is hundreds and thousands of times larger than that of a conventional optical fiber and a plasmon resonance cell. This improves the sensitivity of the method and the speed of the intermolecular interaction. Currently, photonic crystal fibers are considered some of the most promising elements of waveguide optical sensors. Their main advantages include immunity from exposure to electromagnetic fields, high sensitivity and reliability, reproducibility and a wide dynamic range of measurements, the possibility of spectral and spatial multiplexing of sensitive elements, a short response time to changes in the measured value (1 min), a small sample volume, and a small size.

Various modifications, achievements of photonic crystal biosensors and the possibilities of their use for bacterial detection are described in [148,149,150]. Despite the inherent attractiveness of photonic methods, alternative approaches are based on the effect of an electric field on the suspension under study.

## 5. Optical Sensor Systems Based on Measurement of Orientational Effects

Measuring variations in the optical properties of a suspension during the orientation of anisotropic cells has at least two applications. In the first area, the study of the relaxation part of a signal in the analysis of the orientation of cells under some physical influence and their disorientation can be used directly to determine the cell size. For orientation/disorientation, the start–stop mode of movement of the suspension as a flat film or the start–stop mode of the suspension in a cylindrical layer is used. In either case, this type of fluid movement creates a hydrodynamic effect of Tailor–Quette orientation [151,152]. The orientation and disorientation of cells are described by relaxation curves, in the form of which a rotational diffusion coefficient is manifested, depending on the size and geometry of the cells.

The undeniable advantage of this method for the morphometric validation of biotechnological processes is the absence of need for sample preparation during measurements.

The second area of application of the orientation processes of anisotropic cells is their use as an integral part of electro-optical measurements, which will be discussed below.

### 5.1. Optical Sensor Systems Based on Measurements of Bacterial Electrical Characteristics

Yet another direction in the development of optical sensors is their use to determine the electrical properties of cellular structures. The probing effect that causes cells to manifest electrophysical properties is an alternating electric field. Under its influence, electrical charges appear at the boundaries of contact between cellular structures. Their magnitude and sign depend on the complex dielectric properties of the adjacent cellular structures.

The electric properties of a sample of any substance are described by the complex dielectric permittivity ἑ:

ἑ = ***σ*** + 1/j***ω***ε (1)

where ***σ*** is the specific conductivity of a substance, ***ω*** is the electric field frequency, and ε is the dielectric permittivity.

For example, the magnitude of the induced charges at the boundary between the membrane and the cytoplasm is determined by the function

***F***((ε_c_ + ***σ***_c_/j***ω***)/(εm + ***σ***_m_/j***ω***)) (2)

where ε_c_ and ε_m_ are the respective dielectric permittivities of the cytoplasm and the membrane, ***σ***_c_ and ***σ***_m_ are the specific electric conductivities of the cytoplasm and the membrane, and ***ω*** is the frequency of the electric field *E*.

The integral effect of the summation of the induced charges will be a superposition of the functions ***F_i_*** over all contacting cellular structures [153].

Interaction of the induced charges with an electric field gives rise to a torque (an ordered change in cellular orientation and a change in the optical properties of the suspension). Because complex influence of the electric field frequency on the magnitude of the induced charges is present in the *F*-type functions, the change in the optical properties will have frequency dispersion. This, ultimately, makes it possible to ascertain correspondence between changes in the optical properties of the suspension and some electrophysical variables of cellular structures [154]. Depending on the type of measurement and the electric field frequency, the integral specific conductivity of the cellular sample or its dielectric constant can be determined by using the probing action. Accordingly, these will be the methods of high-frequency conductometry or dielectrometry.

Depending on the spatial structure of the electric field, the electric field vector may be in a stationary or switched position parallel or orthogonal to the direction of the light flux. In the first case, one measures the change in the absorbance of the suspension owing to the transition of cells from random orientation to that in the direction of the field. In the second case, an electrorotational effect of cell rotation occurs [155]. The dielectric model for bacterial cells, the mechanisms of cell polarization under the effect of an electric field, and the capabilities of microfluidic sensor systems for identifying bacteria were described in detail in [156].

#### 5.1.1. Electrorotational Sensors

**Figure 17:**
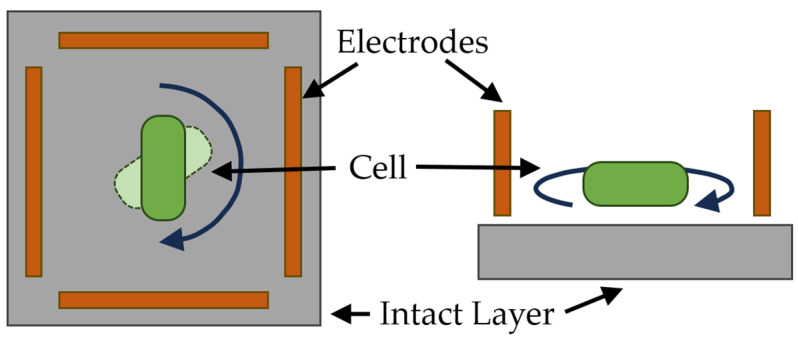
General diagram of electrorotational technology.

The essence of electrorotational analysis is that a rotating electric field is created by four electrodes in a square arrangement, fed by four signals of equal amplitude and frequency, with phase shifts of 0°, 90°, 180° and 270°, respectively. The general diagram of electrorotational technology is shown in Figure 17.

Various modifications to the method have been proposed on the basis of both direct microscopic control of electrorotation and light-scattering methods [157]. To unambiguously determine the rotation of bacterial cells with direct microscopic control, one needs a microscope with high resolution and a small working distance. Therefore, an extremely thin polypropylene spacer with a height of 0.2 mm is installed on the electrodes and the glass substrate. The installation of a microscope on an electrorotational device was described in [158], and the theoretical and experimental significance of the electrorotational technique was described in [153,156,157,158,159]. Electrorotation makes it possible to accurately determine changes in the cytoplasm and membranes [159,160,161,162]. Dielectrophoresis and electrorotation have been developed for the analysis of individual cells [156,158,162,163,164]. However, electrorotation has serious disadvantages, including that measurements are made on single cells and that reliable results can be produced only after a large number of parallel experiments.

#### 5.1.2. Electro-Optical Sensors with a Fixed Direction of the Electric Field Vector

Electro-optical analysis, based on the study of cells as electrophysical objects with a layered structure and on the measurement of the polarization characteristics of cellular structures, is a new approach to assessing the vital physiological characteristics of cells and their heterogeneity. The method is based on using particle polarizability in an electric field and measuring the optical manifestation of the polarizability results.

The electric analysis of cells, supplemented with optical recording, allows selective analysis of the electric conductivity of the cell cytoplasm and other variables of cellular structures. Different versions of electro-optical analysis are based on a chain of the following physical processes. The probing electric field gives rise to induced charges (polarization) at the cytoplasm–membrane–cell wall–external low-conducting medium interfaces. The interaction of these charges with the field that generated them leads to partial orientation of the cells. The change in orientation is recorded optically by the change in light scattering, optical turbidity, or light birefringence.

The cell polarizability tensor can be calculated for a cell structure by using electrodynamic equations. The electro-optical signal is defined, up to constants, as the difference between the longitudinal and transverse components of this tensor [165,166].

Actually, the electrophysical model for the cell, connecting the electrophysical properties and morphometry of cellular structures with the cell polarizability tensor, provides a way to solve inverse problems of restoring the required variable from the measured frequency dispersion of the electro-optical signal.

In most cases, it is not the absolute value of the variable that is of interest but its change during cell growth or various physicochemical effects.

**Figure 18:**
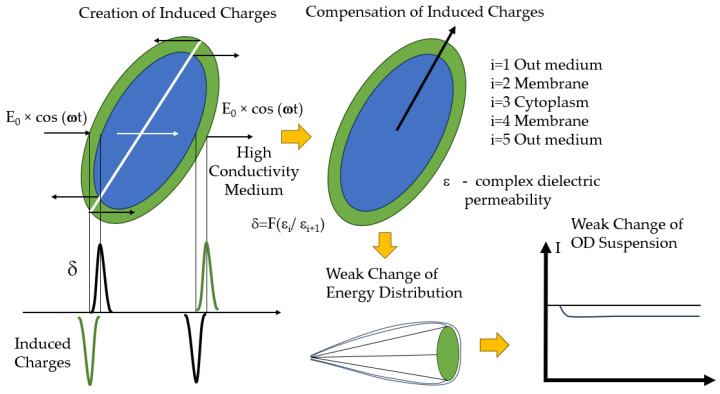
Main mechanisms of electro-optical analysis.

The low-frequency portion of the frequency dispersion of the electro-optical signal is associated with changes in the surface properties of cells (Figure 18). It is used to detect changes in cells after exposure to monoclonal, polyclonal, and phage antibodies in the range of cell densities being detected 10^8^–10^4^ cells mL^−1^ [167,168,169].

The frequency dispersion and absolute value of the signal before any effect serves as a standard for evaluating changes in the state of the cell surface.

The frequency dispersion of the electro-optical signal in the frequency range of approximately 50 to 800 kHz directly reflects the specific value of the cell cytoplasm. The high-frequency portion of the frequency dispersion of the signal makes it possible to determine the specific electrical conductivity of the cell membrane and the efficiency of transport processes when cell growth is monitored [170].

As in the case of hydrodynamic particle orientation, the electro-optical signal of cell disorientation after termination of the electric field represents a relaxation curve. Its shape can be used to determine the distribution of cell sizes in a population. But in batch cultivation, as established experimentally, it is sufficient to determine the average cell size. The shape of the size distribution remains unchanged, but the sizes of all age cell groups change synchronously.

In a certain sense, electro-optics complements and expands the capabilities of the optical method for analyzing cellular structures with different refractive indices. From polarizability theory, it follows that the value of the refractive index for a substance in the optical range corresponds to the asymptotic behavior of the real part of the value of the complex dielectric constant. Electrophysical analysis makes it possible to additionally analyze the imaginary part of the complex dielectric permittivity, called the electric conductivity of the medium, and to use the functional dependence of the change in these variables on the electric field frequency.

The main disadvantage of electro-optical measurements is the sample preparation process. Cells must be transferred to a medium with a specific electrical conductivity below 10–15 mS cm^−1^. Only in this case do the induced charges at the cell surface–membrane–cytoplasm interface remain unsuppressed by the high induced charge of another sign at the interface between the external environment and the cell surface.

## 6. Conclusions

Biosensors are a good alternative to classical techniques. According to Markets and Markets, the biosensor market was valued at USD 25.5 billion in 2021 and is forecast to reach USD 36.7 billion by 2026 [171,172]. Recently, there has been a growing trend towards the use of smartphone-based optical bacterial analysis systems (smartphone-based photo- and spectrometers and smartphone-based fluorimeters [66,173]. The use of optical biosensors may strongly increase the possibility of early pathogen detection and, as a result, may facilitate disease screening. Of the available detection methods, optical biosensors offer many advantages, especially in terms of increased analytical speed. Optical biosensors instantaneously provide interactive information through real-time analysis. 

Developing a biosensor with the necessary properties for reliable and efficient use in everyday applications is an important task for researchers, because in bacterial detection, it is difficult to meet standards. Fluorescence and SERS sensors are the ones developed most actively for the design of sensor systems for the sensitive, selective and reproducible detection of bacteria and their metabolites as markers for diagnosing a wide range of diseases [174].

This review has described different types of optical biosensors and their application for bacterial detection. The biosensors are diverse, as each one is designed according to the needs and objectives of a specific application. It should be noted that each method presented has advantages and disadvantages, which are summarized in Table 1.

An important direction in current research on optical sensor systems is to make electro-optical platforms based on the effect of an electric field on a cell suspension, without changing the vital biochemical and physiological characteristics of cells.

Recent advances allow optical biosensors to be considered promising diagnostic tools that combine the speed of detection of specific molecular markers, simplicity, user-friendliness, efficiency, accuracy, and cheapness with the tendency to make portable platforms. These qualities exceed the generally accepted standards for microbiological and immunological diagnostics and open up broad prospects for the use of these analytical systems in clinical practice directly at the point of care. In the design of new biosensors for practical applications, properties such as reliability, reproducibility, simplicity, and shelf life should be considered. Further standardization and automation of optical sensor systems will expand the range of their application in microbiology, biotechnology, veterinary medicine, medicine, and environmental protection.

## Figures and Tables

**Table 1:** Main advantages and disadvantages of optical sensor systems for bacterial detection.

Technique	Principle	Advantage	Disadvantage
Colorimetry	Determination of the substance concentration by the color intensity of solutions (by the absorption of light by solutions).	- Simplicity. - Efficiency. - Fast analysis. - Customizable array for specific analytes. - Responsiveness to a wide range of analytes. - Flexibility owing to integration with smartphone technology - Small sample size.	- Occurrence of a subjective error and low accuracy. - Problem of dye biocompatibility, reproducibility and visualization. - Difficulty in identifying individual components of a mixture. - Stability/shelf life. - Large data sets for analysis.
Fluorescence	Short-term absorption of a light quantum by a fluorophore (a substance capable of fluorescing), followed by rapid emission of another quantum (which has properties different from the original one).	- Simple design and versatility. - High specificity. - Ability to measure analyte concentrations by fluorescence intensity and decay time. - Immunity to light scattering.	- The need to label samples with fluorescent reagents, which increases the time and cost. - Potential toxicity of reagents. - Vulnerability to interference and autofluorescence. - Short fluorophore lifetime, photostability and loss of recognition capability.
Surface plasmon resonance (SPR)	An optical phenomenon that can be used to monitor interactions between biomolecules in their natural state in real time. It is based on changes in the direction of propagation of a light flux through an optical fiber or a triangular prism coated with a thin metal film	- Possibility of conducting research in real time. - High throughput and specificity. - Suitability for use in harsh environments and resistance to corrosion. - Possibility of integration with fiber optics.	- Difficulty in distinguishing between specific and nonspecific adsorption. - Limited penetration of bacteria by the electromagnetic field and the similarity in the refractive index between the bacterial cytoplasm and the aqueous medium. - Bulky size of the SPR prism. - The need to develop a special algorithm for the smartphone-based approach.
Surface-enhanced Raman scattering (SERS)	The molecule being measured is adsorbed on the surface of a rough nanometallic material, and the Raman signal from the material being measured is enhanced.	- High sensitivity and wide application area. - Less sensitivity to temperature changes and water. - Measurements can be made by using opaque substrates. Suitability for any surface owing to diffuse light	- Many complex phenomena observed in the experiment cannot be explained by the existing SERS theory. - Possible interference from other molecules, fluorescence and turbidity. - Unstable wavelength and laser intensity. - Long preparation time. - Problems with fluorescent or highly absorbing materials.
Fiber Optic Sensors	Use of the property of total internal reflection of the wind when it passes through the waveguide and creates a boundary of evanescent waves on the surface of the waveguide.	- Sensitivity. - Slow response time (1–3 min). - Non-contact nature and low influence of electrical interference. - Possibility of detection bacteria without the use of specific molecules (by the spectral characteristics of pathogens). - Ease of use and small size. - By using a sandwich format, waveguide biosensors have been developed to analyze dirty samples such as *B. anthracis* spores mixed with powders with little sample pretreatment [175]. - Portable automated systems such as RAPTOR have undergone rigorous field testing.	- Sensitivity of traditional fiber-optic sensors is very limited owing to the confinement of light in the fiber core, so the interaction between light and analyte is very weak. - The birefringent fiber used, for example, for sensing in the fiber-optic sagnac interferometer probe, is very sensitive to temperature [130]. - Possibility of nonspecific binding of analyte cells on the waveguide surface.
Optical Sensors Based on Ionophores	A combination of measurements on the basis of vanishing field sensing and optical phase difference. The interference signal produced by interfering fields is detected at the sensor output, and the signal is related to the analyte concentration.	- Requires a small amount of sample. - Easy to use.	- Interference from other ions (interfering ion dominates over the primary ion). - Noise level of the instrument. - Loss of sensitivity owing to the sigmoidal shape of the response curve. - Analyte depletion (e.g., measuring a change in the analyte concentration in a sample, response time is too long).
Photonic Crystal Biosensors	The operating principle of photonic crystal waveguides is based on the detection and identification of biological objects by using the spectra of light passing through a hollow core filled with the material under study in the wavelength range 200–1100 nm.	- Immunity from electromagnetic fields. - High sensitivity and reliability of the method. - Reproducibility and wide dynamic range of measurements. - Possibility of spectral and spatial multiplexing of sensitive elements. - Short response time to changes in the measured value (1 min), small sample volumes, small size. - Possibility of integration with new technologies (smartphones) for use as a potential diagnostic tool.	- Preparation steps are necessary (e.g., filtering). - Background interference is possible.
Optical sensor systems based on measurement of orientational effects	The probing effect of an electrical field causes electrical charges to appear at the boundaries of contact between cellular structures. Their magnitude and sign depend on the complex dielectric properties of the adjacent cellular structures.	- Integrated analysis of electrophysical variables of cellular structures. - Possibility to trace metabolic changes in microbial cells. - High sensitivity. - Small sample volume. - Short response time (5–20 min) - Possibility of integration with new technologies (smartphones) for use as a potential diagnostic tool	- The need to conduct research under conditions of reduced conductivity of the measurement environment.

## Data Availability

Not applicable.
